# Supplementary material for: Propagation and Diffusion of Fluorescent Substances with Footprints in Indoor Environments
Source: Int J Environ Res Public Health. 2022 Jun 24;19(13):7733. doi: 10.3390/ijerph19137733 (PMC9266062; doi:10.3390/ijerph19137733)
Supplement: Supplementary file 1 [file ijerph-19-07733-s001.zip › ijerph-1733747-supplementary.pdf]

Supporting information

Propagation and diffusion of fluorescent substances with footprints in indoor environments

Comments

Figure S1: Photographic images of the experimental female (left) and male (right) shoes.

Figure S2: Gray fluorescence images of the contaminated floor panels for single-footprint walking.

Table S1: Normalized gray value of the fluorescent solution with the number of walking steps.

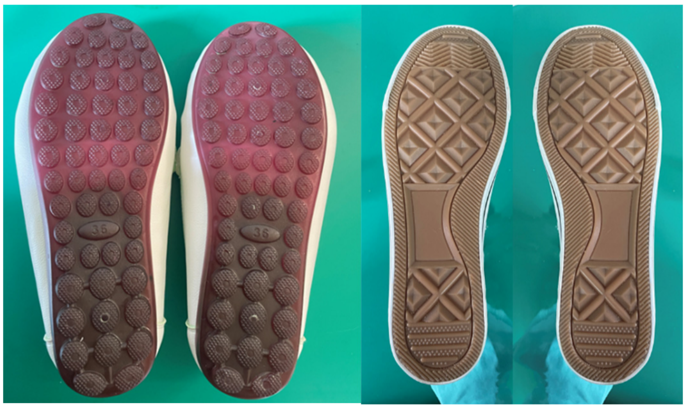

Figure S1: Photographic images of the experimental female (left) and male (right) shoes.

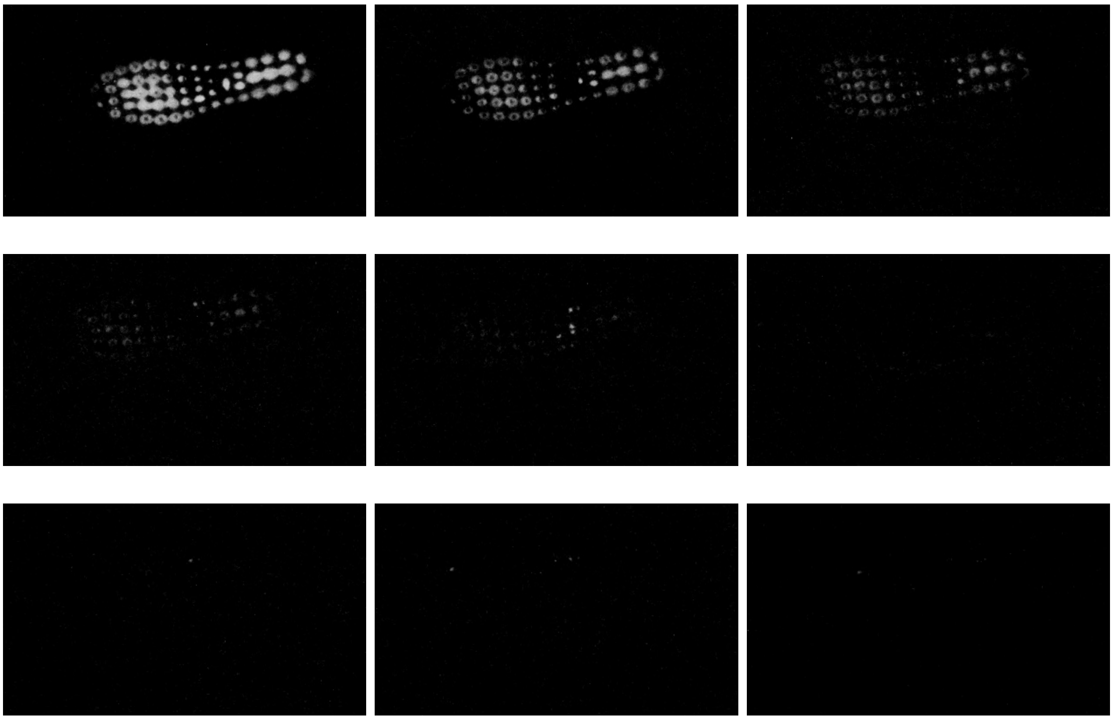

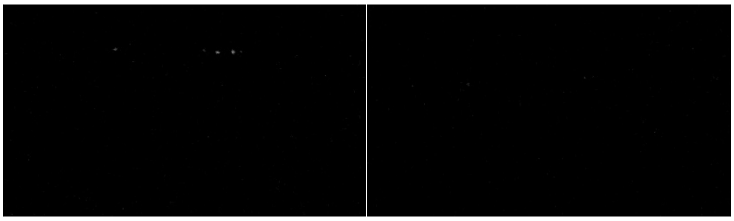

Figure S2: Gray fluorescence images of the contaminated floor panels for single-footprint walking.

Table S1: Averaged normalized gray values of the fluorescent solution with the number of walking steps.

The number of steps	50-80-W	50-100-O	50-120-O	60-80-O	60-100-W	60-120-W	70-80-O	70-100-W	70-120-W
1	1	1	1	1	1	1	1	1	1
2	0.25883	0.39967	0.44424	0.36911	0.41395	0.34049	0.41653	0.38466	0.47186
3	0.07186	0.10203	0.15077	0.11911	0.18421	0.15772	0.18401	0.22924	0.20561
4	0.0279	0.01825	0.03159	0.05511	0.09528	0.06611	0.10053	0.10497	0.10613
5	0.01503	0.01119	0.01379	0.03627	0.03894	0.03731	0.0545	0.05384	0.05891
6	0.01192	0.0086	0.01416	0.02799	0.02871	0.02742	0.04152	0.03074	0.04367
7	0.00755	0.00915	0.01188	0.02544	0.01921	0.01702	0.0353	0.02567	0.02994
8	0.00256	0.01392	0.01004	0.02165	0.01037	0.0147	0.029	0.01795	0.02475
9	0.00858	0.01309	0.00867	0.01598	0.00625	0.01378	0.02485	0.01353	0.02371
10	0.00278	0.01011	0.00839	0.01274	0.00806	0.00842	0.02197	0.0125	0.02055
11	0.01045	0.00896	0.00799	0.01234	0.00866	0.01066	0.01619	0.01167	0.01505
